# Evaluation of Serum Des-Gamma-Carboxy Prothrombin for the Diagnosis of Hepatitis B Virus-Related Hepatocellular Carcinoma: A Meta-Analysis

**DOI:** 10.1155/2018/8906023

**Published:** 2018-10-04

**Authors:** Jiu Chen, Guolin Wu, Youdi Li

**Affiliations:** Department of Traditional Chinese Medicine, The First Affiliated Hospital, College of Medicine, Zhejiang University, #79 Qingchun Road, Hangzhou, Zhejiang Province, China 310003

## Abstract

**Aim:**

To explore the diagnostic efficacy of des-gamma-carboxy prothrombin (DCP) in hepatitis B virus- (HBV-) related hepatocellular carcinoma (HCC).

**Methods:**

A retrospective study of 459 cases from June 2016 to March 2018 was undertaken, and records of the DCP levels were extracted. The sensitivity, specificity, and cutoff points were calculated using SPSS 17.0 software. A systematic search in PubMed, Web of Science, and the Cochrane Central Register of Controlled Trials was performed for articles published in English from 1997 to 2017, focusing on serum DCP for HBV-related HCC. Data on sensitivity, specificity, the positive likelihood ratio (PLR), negative likelihood ratio (NLR), and diagnostic odds ratio (DOR) were extracted from five studies by systematic search and one study of our own. The summary receiver operating characteristic (sROC) curve was obtained, and the area under the receiver operating characteristic (AUROC) curve was calculated.

**Results:**

The pooled sensitivity, specificity, PLR, NLR, and DOR were 0.71 (95% CI: 0.59, 0.80), 0.93 (95% CI: 0.87, 0.96), 9.5 (95% CI: 5.2, 17.5), 0.32 (95% CI: 0.22, 0.46), and 30 (95% CI: 13, 72), respectively. The AUROC curve was 0.91 (95% CI: 0.88, 0.93).

**Conclusions:**

In the diagnosis of HBV-related hepatocellular carcinoma (HCC), DCP is an ideal marker that should be considered for surveillance purposes.

## 1. Introduction

Primary liver cancer is the fifth most common cancer and is responsible for the second most common cancer-related deaths worldwide [1]. Hepatocellular carcinoma (HCC) alone accounts for approximately 80% of all cases [2] and is one type of malignancy with a very poor prognosis [1]. Early identification can improve the prognosis. However, HCC usually arises against the background of liver damage, and the tumors are usually relatively large before any symptoms become evident [3]. Previous studies have shown that if patients are diagnosed at an early stage, the 5-year survival rate can be above 70% [4]. If patients are diagnosed at a late stage, however, the 5-year survival rate is less than 5% [5]. These features make early identification both difficult and important.

Ultrasonography (US) alone, without concurrent detection of serum alpha fetoprotein levels, has been recommended for the surveillance of HCC, according to the representative guidelines of the American Association for the Study of Liver Diseases in 2010 [6] and the European Association for the Study of the Liver in 2012 [7]. However, interpretation of the sonogram is dependent on the expertise of the operator and quality of the equipment [8] and can be disrupted by other conditions of the liver, such as cirrhosis [9]. The overall sensitivity of US in this context is only 0.593 [10].

The identification of new markers for the diagnosis of HCC is urgently required. Serum des-gamma-carboxy prothrombin (DCP), also known as prothrombin-induced by vitamin K absence-II (PIVKA-II), was first reported by Liebman et al. in 1984 [11]. Its value has been confirmed in the diagnosis of HCC in a series of clinical trials [12–20]. However, the sensitivity, specificity, and cutoff points in previous studies have been inconsistent, and in some instances, even conflicting [21]. One of the reasons for this disparity could be the differences in etiology and the fact that diagnostic values were mainly derived from various patients without homogeneous etiologies.

Hepatitis B virus (HBV) infection is the main causative factor of HCC, and more than half of all cases could be attributed to HBV infection worldwide [22]. Even with improvements in technologies, the morbidity and mortality of HBV-related HCC have still shown a steady increase [23].

Within the context of increased sensitivity, DCP has been considered for the diagnosis, treatment response, recurrence monitoring, and prognosis of HBV-related HCC [24–28]. However, the precise diagnostic efficacy has never been fully evaluated. We conducted this meta-analysis to elucidate this pertinent issue.

## 2. Methods

### 2.1. Retrospective Study

A total of 459 participants were retrospectively enrolled at the First Affiliated Hospital, College of Medicine, Zhejiang University, from June 2016 to March 2018. The study was approved by the Ethics Committees of the First Affiliated Hospital, College of Medicine, Zhejiang University.

#### 2.1.1. Criteria of Selection

1. Patients were hepatitis B surface antigen-positive (>0.05 IU/mL) and/or HBV DNA-positive (>30 IU/mL)
2. Serum DCP and indexes of HBV were both measured at the same time
3. The DCP was measured by enzyme immunoassay
4. The diagnosis of HCC was based on histological examination

#### 2.1.2. Criteria of Exclusion

1. The diagnosis of HCC was based on imaging characteristics
2. Patients were also affected by other types of hepatitis virus infections, nonalcoholic steatohepatitis, alcoholic steatohepatitis, drug-induced hepatitis, or autoimmune hepatitis
3. Patients had undergone liver transplantation
4. Patients were taking vitamin K or warfarin within one week of DCP measurement
5. Patients showed other evidence of other kinds of malignancies besides HCC
6. Nodules in the liver could not have been confirmed; for example, the patients refused biopsy or surgery
7. Patients were treated before DCP measurement

### 2.2. Literature Screening for Meta-Analysis

Considering that the diagnostic technique was revised in 1997, resulting in a much higher sensitivity [29], a systematic search was conducted from January 1, 1997, to December 31, 2017, by two investigators independently (Jiu Chen and Youdi Li). Searches for relevant studies were mainly conducted in PubMed, Web of Science, and the Cochrane Central Register of Controlled Trials.

**Figure 1:**
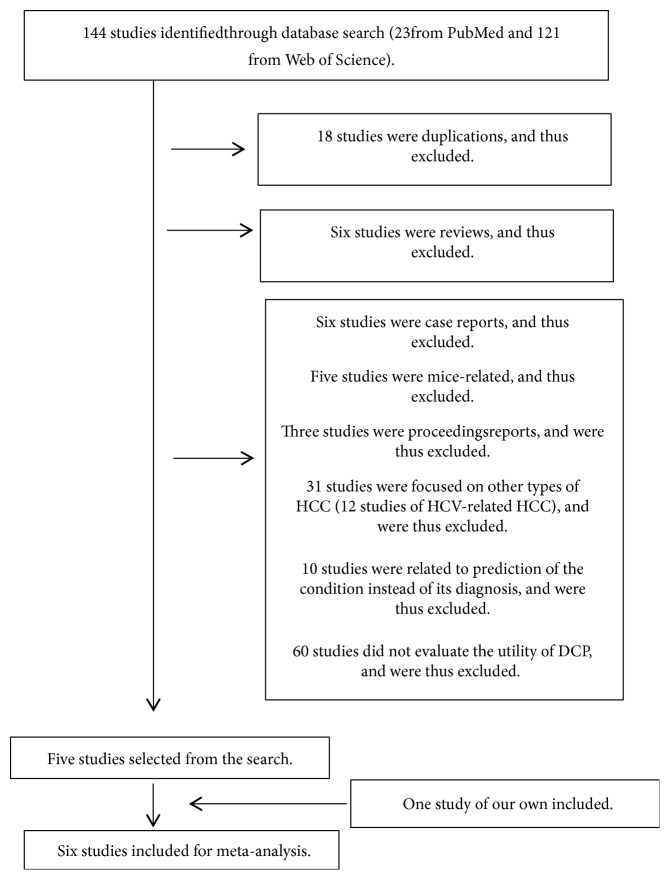
Literature screening was performed at four levels. Flowchart of literature research and study selection.

The literature search strategy is summarized and depicted in Table 1. A flow diagram of the study selection process is summarized in Figure 1.

#### 2.2.1. Levels of Literature Screening

Literature screening was performed at four levels, as follows.

*(1) Level 1*. Level 1 was derived from the three databases.

(i) PubMed
(((((((((((((((((((des-gamma-carboxyprothrombin [Title/Abstract] OR ((des-gamma-carboxy [All Fields] AND “prothrombin”[MeSH Terms]) OR des-gamma-carboxy prothrombin [Title/Abstract])) OR ((des[All Fields] AND gamma-carboxy[All Fields] AND “prothrombin”[MeSH Terms]) OR des[All Fields] AND gamma-carboxy[All Fields] AND prothrombin [Title/Abstract])) OR Isoprothrombin [Title/Abstract]) OR prothrombin precursor [Title/Abstract]) OR PIVKA [Title/Abstract]) OR (((“proteins”[MeSH Terms] OR “proteins”[All Fields] OR “protein”[All Fields]) AND induced [All Fields] AND (“vitamin k”[MeSH Terms] OR “vitamin k”[All Fields]) AND absence [All Fields]) OR (protein induced by vitamin K absence [Title/Abstract] OR antagonists [Title/Abstract]))) OR ((descarboxylated [All Fields] AND “prothrombin”[MeSH Terms]) OR descarboxylated prothrombin [Title/Abstract])) OR ((acarboxy [All Fields] AND “prothrombin”[MeSH Terms]) OR acarboxy prothrombin [Title/Abstract])) OR (((“proteins”[MeSH Terms] OR “proteins”[All Fields] OR “protein”[All Fields]) AND induced [All Fields] AND (“vitamin k”[MeSH Terms] OR “vitamin k”[All Fields]) AND absence [All Fields]) OR (protein induced by vitamin K absence [Title/Abstract] OR antagonist-II [Title/Abstract]))) OR PIVKA-II [Title/Abstract]) OR PIVKA II [Title/Abstract]) OR ((non-carboxylated [All Fields] AND “prothrombin”[MeSH Terms]) OR (non-carboxylated[All Fields] AND factor II[Title/Abstract]))) OR descarboxyprothrombin[Title/Abstract]) OR des-gamma-carboxyprothrombin [Title/Abstract]) OR ((des-gamma-carboxy [All Fields] AND “prothrombin”[MeSH Terms]) OR des-gamma-carboxy prothrombin [Title/Abstract])) OR ((des [All Fields] AND gamma-carboxy [All Fields] AND “prothrombin”[MeSH Terms]) OR des [All Fields] AND gamma-carboxy [All Fields] AND prothrombin [Title/Abstract])) OR decarboxyprothrombin [Title/Abstract]) OR DCP [Title/Abstract]) AND ((((((“hepatitis b virus”[MeSH Terms] OR (Particle [All Fields] AND Dane [Title/Abstract])) OR (“hepatitis b virus”[MeSH Terms] OR Dane Particle[Title/Abstract])) OR (“hepatitis b virus”[MeSH Terms] OR ((“hepatitis viruses”[MeSH Terms] OR (“hepatitis”[All Fields] AND “viruses”[All Fields]) OR “hepatitis viruses”[All Fields] OR (“hepatitis”[All Fields] AND “virus”[All Fields]) OR “hepatitis virus”[All Fields]) AND Homologous Serum[Title/Abstract]))) OR (“hepatitis b virus”[MeSH Terms] OR ((“virology”[Subheading] OR “virology”[All Fields] OR “viruses”[All Fields] OR “viruses”[MeSH Terms]) AND Hepatitis B[Title/Abstract]))) OR (“hepatitis b virus”[MeSH Terms] OR Hepatitis B viruses[Title/Abstract])) OR (“hepatitis b virus”[MeSH Terms] OR B virus, Hepatitis[Title/Abstract]))) AND (((((((((((((((“carcinoma, hepatocellular”[MeSH Terms] OR Hepatomas[Title/Abstract]) OR (“carcinoma, hepatocellular”[MeSH Terms] OR Hepatoma[Title/Abstract])) OR (“carcinoma, hepatocellular”[MeSH Terms] OR Hepatocellular Carcinoma[Title/Abstract])) OR (“carcinoma, hepatocellular”[MeSH Terms] OR Liver Cell Carcinomas[Title/Abstract])) OR (“carcinoma, hepatocellular”[MeSH Terms] OR ((“cells”[MeSH Terms] OR “cells”[All Fields] OR “cell”[All Fields]) AND (“carcinoma”[MeSH Terms] OR “carcinoma”[All Fields] OR “carcinomas”[All Fields]) AND Liver[Title/Abstract]))) OR (“carcinoma, hepatocellular”[MeSH Terms] OR ((“cells”[MeSH Terms] OR “cells”[All Fields] OR “cell”[All Fields]) AND Carcinoma, Liver[Title/Abstract]))) OR (“carcinoma, hepatocellular”[MeSH Terms] OR ((“carcinoma”[MeSH Terms] OR “carcinoma”[All Fields] OR “carcinomas”[All Fields]) AND Liver Cell[Title/Abstract]))) OR (“carcinoma, hepatocellular”[MeSH Terms] OR Carcinoma, Liver Cell[Title/Abstract])) OR (“liver neoplasms”[MeSH Terms] OR ((“neoplasms”[MeSH Terms] OR “neoplasms”[All Fields] OR “cancers”[All Fields]) AND Liver[Title/Abstract]))) OR (“liver neoplasms”[MeSH Terms] OR Cancer, Liver[Title/Abstract])) OR (“liver neoplasms”[MeSH Terms] OR Liver Cancers[Title/Abstract])) OR (“liver neoplasms”[MeSH Terms] OR Liver Cancer[Title/Abstract])) OR (“carcinoma, hepatocellular”[MeSH Terms] OR Liver Cell Carcinoma[Title/Abstract])) OR (“carcinoma, hepatocellular”[MeSH Terms] OR Hepatocellular Carcinomas[Title/Abstract])) OR (“carcinoma, hepatocellular”[MeSH Terms] OR ((“carcinoma”[MeSH Terms] OR “carcinoma”[All Fields] OR “carcinomas”[All Fields]) AND Hepatocellular[Title/Abstract]))) AND ((“1997/01/01”[PDAT]: “2017/12/31”[PDAT]) AND “humans”[MeSH Terms] AND English[lang])
Altogether, 23 articles were included
(ii) Web of Science
Filters: Index=SCI-EXPANDED, SSCI, A&HCI, CPCI-S, CPCI-SSH, ESCI, CCR-EXPANDED, IC Time limited=1997-2017
  Key words: (Carcinomas, Hepatocellular) OR Key words: (Hepatocellular Carcinomas) OR Key words: (Liver Cell Carcinoma) OR Key words: (Liver Cancer) OR Key words: (Liver Cancers) OR Key words: (Cancer, Liver) OR Key words: (Cancers, Liver) OR Key words: (Carcinoma, Liver Cell) OR Key words: (Carcinomas, Liver Cell) OR Key words: (Cell Carcinoma, Liver) OR Key words: (Cell Carcinomas, Liver) OR Key words: (Liver Cell Carcinomas) OR Key words: (Hepatocellular Carcinoma) OR Key words: (Hepatoma) OR Key words: (Hepatomas)
  Key words: (B virus, Hepatitis) OR Key words: (Hepatitis B viruses) OR Key words: (viruses, Hepatitis B) OR Key words: (Hepatitis Virus, Homologous Serum) OR Key words: (Dane Particle) OR Key words: (Particle, Dane)
  Key words: (DCP) OR Key words: (decarboxyprothrombin) OR Key words: (des(gamma-carboxy)prothrombin) OR Key words: (des-gamma-carboxy prothrombin) OR Key words: (des-gamma-carboxyprothrombin) OR Key words: (descarboxyprothrombin) OR Key words: (non-carboxylated factor II) OR Key words: (PIVKA II) OR Key words: (PIVKA-II) OR Key words: (protein induced by vitamin K absence or antagonist-II) OR Key words: (acarboxy prothrombin) OR Key words: (descarboxylated prothrombin) OR Key words: (protein induced by vitamin K absence or antagonists) OR Key words: (PIVKA) OR Key words: (prothrombin precursor) OR Key words: (Isoprothrombin) OR Key words: (des(*γ*-carboxy)prothrombin) OR Key words: (des-γ-carboxy prothrombin) OR Key words: (des-γ-carboxyprothrombin)
  (3) AND (2) AND (1): limited in the English language

Altogether, 137 studies were included. Two studies were excluded because they were not written in English, one was excluded because of duplication, four more were excluded because they reported on the proceedings of various conferences, and nine were excluded because they were reviews. Thus, 121 articles were included after the first level screening.

(iii) Cochrane Central Register of Controlled Trials
  (Carcinomas, Hepatocellular) or (Hepatocellular Carcinomas) or (Liver Cell Carcinoma) or (Liver Cancer) or (Liver Cancers) or (Cancer, Liver) or (Cancers, Liver) or (Carcinoma, Liver Cell) or (Carcinomas, Liver Cell) or (Cell Carcinoma, Liver) or (Cell Carcinomas, Liver) or (Liver Cell Carcinomas) or (Hepatocellular Carcinoma) or (Hepatoma) or (Hepatomas)
  (B virus, Hepatitis) or (Hepatitis B viruses) or (viruses, Hepatitis B) or (Hepatitis Virus, Homologous Serum) or (Dane Particle) or (Particle, Dane)
  (DCP) or (descarboxyprothrombin) or (des gamma-carboxy prothrombin) or (des-gamma-carboxy prothrombin) or (des-gamma-carboxyprothrombin) or (decarboxyprothrombin) or (non-carboxylated factor II) or (PIVKA II) or (PIVKA-II) or (protein induced by vitamin K absence or antagonist-II) or (acarboxyl prothrombin) or (decarboxylated prothrombin) or (protein induced by vitamin K absence or antagonists) or (PIVKA) or (prothrombin precursor) or (Isoprothrombin) or (des *γ*-carboxy prothrombin) or (des-γ-carboxy prothrombin) or (des-γ-carboxyprothrombin)
  (1) and (2) and (3)

Altogether, 50 studies were included. 42 studies were reviews, and eight studies were unrelated, after screening of the title and abstract. Thus, no articles were included after the first-level screening.

*(2) Level 2*. Altogether, 144 studies were included and 18 duplications were excluded.

*(3) Level 3*. Altogether, 126 studies were included and six studies were excluded because they comprised reviews.

*(4) Level 4*. Five studies were eventually included from the searches conducted. Six studies were excluded because they were case reports; five studies were excluded because they were mice-related; three studies were excluded because they reported the proceedings of various conferences; 31 studies were excluded because they reported on other types of HCC (12 studies of hepatitis C virus- (HCV-) related HCC); 10 studies were excluded because they were related to the prediction of the condition, instead of its diagnosis; and 60 studies were excluded because they did not evaluate the utility of DCP.

#### 2.2.2. Criteria of Selection for Meta-Analysis

Studies were included if they met the following inclusion criteria:

1. Clinical studies that evaluated the efficiency of DCP for the diagnosis of HBV-related HCC, with a prospective or retrospective design. The efficiency of DCP was assessed either alone or in comparison with other tests
2. The diagnosis of HCC was based on histological examination or the interpretation of appropriate imaging characteristics, as defined by accepted guidelines
3. Selected studies detected the DCP concentration in serum
4. Studies reported both the sensitivity and the specificity of DCP

#### 2.2.3. Criteria of Exclusion for Meta-Analysis

1. Diagnostic criteria and study population were ambiguous
2. Studies detected DCP concentration in tissue and other body fluids
3. Studies evaluated the efficiency of DCP for the prognosis of HCC
4. Studies presented insufficient information to make a judgment
5. Studies were published as reviews, letters, case reports, editorials, or comments
6. Data were excluded in a duplicate publication
7. Studies were not conducted on human subjects

### 2.3. Selection of Studies

The titles and abstracts of all studies derived from the search results were read thoroughly to confirm eligibility, and the full-text versions of all potentially eligible studies were then retrieved for further assessment. Doubts were discussed with a third investigator (Guolin Wu). The authors of the studies were contacted for further details, if necessary.

### 2.4. Data Extraction

We extracted data on the authors, year of publication, country, number of patients, sensitivity, specificity, cutoff points, and area under the receiver operating characteristic (AUROC) curve from the selected studies (Table 2).

### 2.5. Assessment of Methodological Quality

The quality of each study was assessed according to the QUADAS (quality assessment of studies of diagnostic accuracy included in systematic reviews) checklist. Each of the 14 items in the QUADAS checklists was as “yes,” “no,” or “unclear” [30]. Two items were not assessed, one of which was “Were uninterpretable/intermediate results reported?” This question was not assessed because the concentration of DCP was determined by test kits; thus, there were no uninterpretable/intermediate results. The other unassessed item was that of “Withdrawals explained?” This item was not assessed because all the retrospective studies were evaluated without consideration of withdrawals. Twelve items of the QUADAS checklist are shown, and the quality of the studies are presented in Table 3.

### 2.6. Indices of Diagnostic Efficacy

The indices of diagnostic efficacy included sensitivity, specificity, positive likelihood ratio (PLR), negative likelihood ratio (NLR), diagnostic odds ratio (DOR), and summary receiver operating characteristic (sROC) curve.

### 2.7. Data Analysis

For the retrospective evaluation of our previous study, the analysis of the receiver operating characteristic (ROC) curve was applied to measure diagnostic efficacy. The cutoff points and AUROC were calculated using SPSS 17.0 software.

For the meta-analysis, a funnel plot was constructed and *P* values were calculated. Publication bias existed when a *P* < 0.05 was observed. The pooled sensitivity, specificity, PLR, NLR, and DOR were summarized, and the AUROC was calculated. The statistical analyses of the meta-analysis were accomplished, using Stata 13.1 software.

## 3. Results

### 3.1. Characteristic of the Selected Studies

A total of 144 studies were identified from the searches conducted, of which five [9, 24, 31–33] studies were considered suitable for inclusion in the meta-analysis, after exclusion of reviews, case reports, duplications, conference proceedings, and other unsuitable studies. All included studies were published in English. Another suitable study of ours conducted on 459 patients was included. As shown in Table 1, 2472 patients from six studies were eventually included for the meta-analysis.

### 3.2. Quality of the Studies

The results of the QUADAS assessment are presented in Table 2. All included studies adopted a retrospective design, and this was not regarded as the representative of the patient spectrum. In addition, three [24, 31, 32] studies did not clearly describe the exclusion criteria applied. However, other items were effectively reported, and all six studies scored an “A.”

### 3.3. Analysis of Publication Bias

**Figure 2:**
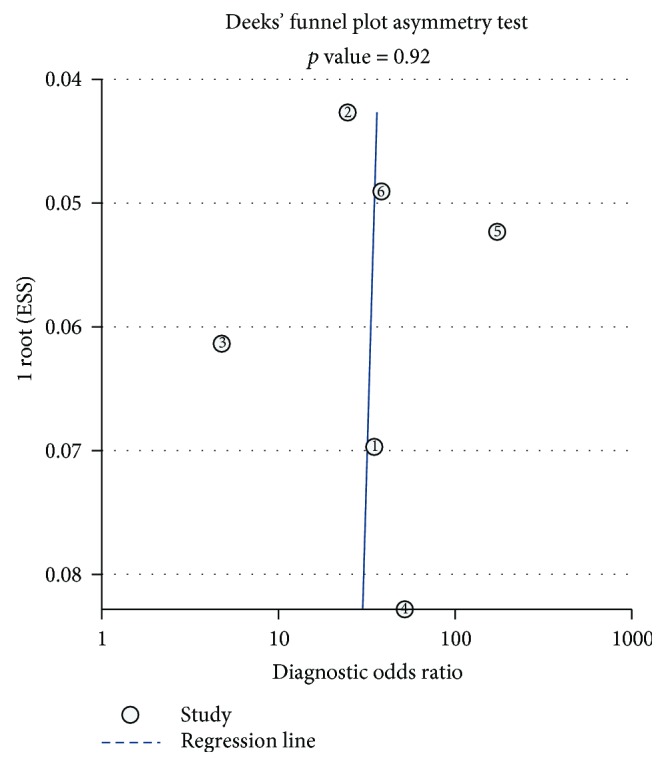
The funnel plot of publication bias. Each dot represented one study. The dots were plotted near the regression line. The funnel plot showed no publication bias among the six studies (*P* = 0.92).

A funnel plot was used to examine publication bias. As shown in Figure 2, the *P* value was 0.92, indicating the absence of publication bias among the six studies.

### 3.4. Heterogeneity Analysis

**Figure 3:**
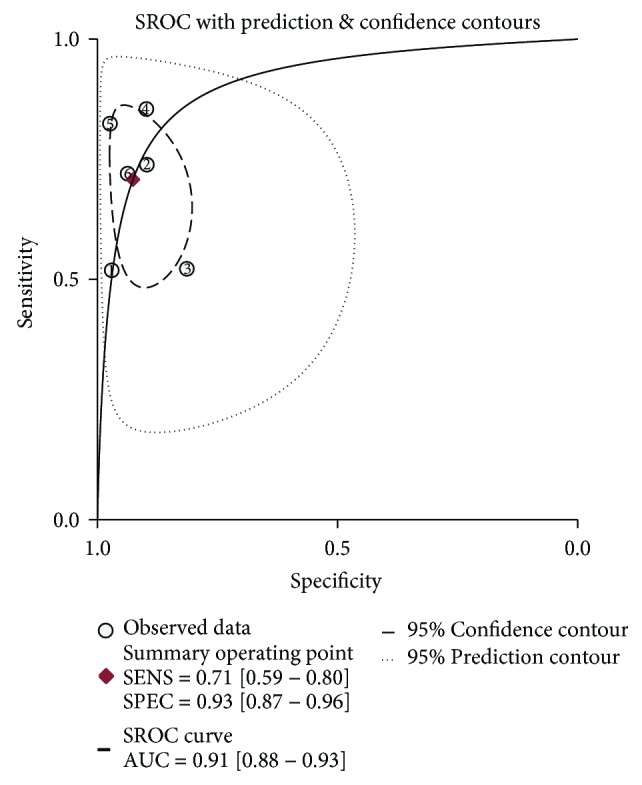
The sROC curve of DCP. Each dot represented one study. The red one represented the summary operating point with the high sensitivity (0.71) and specificity (0.93). The summary receiver operating characteristic (sROC) curve showed excellent diagnostic efficacy with the area under the receiver operating characteristic (AUROC) curve up to 0.91 (close to 1).

The analysis of heterogeneity is presented in Figure 3. The studies could have been incorporated with an index of correlation (mixed model) of 0.33, and the proportion of heterogeneity was likely due to the threshold effect, which was 0.11.

### 3.5. Meta-Analysis

The pooled sensitivity, specificity, PLR, NLR, and DOR were 0.71 (95% CI: 0.59, 0.80), 0.93 (95% CI: 0.87, 0.96), 9.5 (95% CI: 5.2, 17.5), 0.32 (95% CI: 0.22, 0.46), and 30 (95% CI: 13, 72), respectively. The AUROC curve is presented in Figure 3 with a value of 0.91 (95% CI: 0.88, 0.93).

## 4. Discussion

The meta-analysis of the six studies confirmed the diagnostic efficacy of DCP in patients with HBV-related HCC. All six studies were published recently (three being published in 2017). In addition, each of the studies included in the meta-analysis was of high quality and scored an “A” according to the QUADAS checklist. All serum samples were measured by enzyme immunoassay; however, limitations must still be acknowledged. Firstly, all six studies were retrospective, which could increase the representative patient spectrum. Secondly, the cutoff points differed among the studies. The 40 mAU/mL cutoff point in the serum had been established in the population with heterogeneous etiologies [19]. The same cutoff point of 40 mAU/mL was employed in three studies [9, 24, 31] (two from Korea and one from China), whereas the cutoff points of 32.09 mAU/mL [32] and 40.5 m AU/mL [33] were employed in the other two studies (both from China). The study with the lowest cutoff point of 32.09 mAU/mL [32] investigated early-stage HBV-related HCC. In our own study, the cutoff point was 49.05 mAU/mL. This value was the highest among the six studies. One of the main reasons for this relatively high cutoff point was the fact that all HCC cases in our study were diagnosed by histological examination. In comparison with other studies, in which diagnosis was made by histological examination or imaging characteristics, histological examination was considered the gold standard with the highest diagnostic sensitivity and specificity for all cancers' diagnosis [34]. The imaging diagnosis also takes an important role in diagnosis, but sometimes it is discordant with histological examination [35], especially in early HCC [36]. As a general rule, due to the high negative predictive value, reference values defining “below the cutoff point” biomarker concentrations (such as the 99th percentile of a healthy reference population with a coefficient of variation of <10%) are especially useful for diagnostic purposes [37]. With the gold standard of histological examination, the highest cutoff point of our study represents the most accurate level of DCP in HBV-related HCC.

DCP is produced by HCC and can conversely stimulate the growth and invasion of HCC through different signal pathways [38]. The diagnostic efficacy of histological examination has been confirmed for HCC. However, most of the previous studies were conducted in populations with heterogeneous liver diseases, and the respective sensitivities, specificities, and cutoff points were inconsistent among the various studies [21]. The 40 mAU/mL cutoff point has also been confirmed in populations with heterogeneous etiologies predominantly infected with HCV [12–20, 39]. The present study, to our knowledge, is the first to report a meta-analysis that evaluates the diagnostic efficacy of DCP in the detection of HCC with homogeneous etiologies (HBV infection).

The sensitivity, specificity, PLR, NLR, DOR, and AUROC are all indices of diagnostic efficacy. The values of sensitivity, specificity, and the AUROC that are closer to 1 are preferred. A likelihood ratio greater than 1 indicates that the test result is associated with the disease. The value of a DOR ranges from 0 to infinity, and the higher values indicate better diagnostic efficacy. In the present meta-analysis, we included six studies of 2472 HBV-infected patients from China and Korea. We found that in the HBV-infected population, the pooled sensitivity, specificity, PLR, NLR, and DOR were 0.71, 0.93, 9.5, 0.32, and 30, respectively. The AUROC was as high as 0.91. The AUROC of DCP were 0.89, 0.797, and 0.893 in three meta-analyses of predominantly HCV-infected populations [29, 40, 41]. In comparison with the meta-analysis of populations with heterogeneous etiologies, the AUROC value was higher in HBV-related HCC. The diagnostic efficacy of DCP was more favorable in HBV-related HCC.

In various guidelines for HCC worldwide, DCP has only been recommended for the surveillance of HCC. For instance, in the 2013 guidelines of Japan [8], it is recommended for the surveillance of HCC caused mainly by HCV (68%) [42]. The present results show the superior diagnostic efficacy of DCP in HBV-related HCC. In view of these findings, we suggest that DCP should be considered for the surveillance of HCC in the established guidelines of other countries and regions, especially those with a high incidence of HBV infections, such as East Asia (except Japan) and Africa [43].

Ultrasonography has been recommended for the surveillance of HCC in almost all established guidelines worldwide [8]. However, the overall sensitivity of US is only 0.593 [10]. With a sensitivity of 0.71 and specificity of 0.93, DCP seems to be a more favorable choice than US.

With its high diagnostic efficacy, ease of use, and reproducibility, as well as its objectivity and noninvasiveness, DCP is an ideal marker that could be considered an easily accessible complement of US for the surveillance for HBV-related HCC. As this meta-analysis was based on six retrospective studies, more prospective studies with relatively large samples are needed in the future.

## Figures and Tables

**Table 1:** Literature search strategy of the three databases.

Database	Search terms			Search criteria	Filters/limits
	HBV	DCP	HCC		
PubMed	B virus, Hepatitis Hepatitis B viruses viruses, Hepatitis B Hepatitis Virus, Homologous Serum Dane Particle Particle, Dane	DCP (prothrombin) Decarboxyprothrombin des(gamma-carboxy)prothrombin des-gamma-carboxy prothrombin des-gamma-carboxyprothrombin Descarboxyprothrombin non-carboxylated factor II PIVKA II PIVKA- II PIVKA-II (protein induced by vitamin K absence or antagonist-II) acarboxy prothrombin descarboxylated prothrombin PIVKA (protein induced by vitamin K absence or antagonists) PIVKA prothrombin precursor Isoprothrombin des(γ-carboxy)prothrombin des-*γ*-carboxy prothrombin des-γ-carboxyprothrombin	Carcinomas, Hepatocellular Hepatocellular Carcinomas Liver Cell Carcinoma Liver Cancer Liver Cancers Cancer, Liver Cancers, Liver Carcinoma, Liver Cell Carcinomas, Liver Cell Cell Carcinoma, Liver Cell Carcinomas, Liver Liver Cell Carcinomas Hepatocellular Carcinoma Hepatoma Hepatomas	[Title/Abstract] OR [MeSH Terms]	Humans; English Dates: from 1997 to 2017
Web of Science				Key words	Index = SCI-EXPANDED, SSCI, A&HCI, CPCI-S, CPCI-SSH, ESCI, CCR-EXPANDED, IC Time limited = 1997–2017
Cochrane Central Register of Controlled Trials				Key words	NO

HBV (hepatitis B virus), DCP (des-gamma-carboxy prothrombin), HCC (hepatocellular carcinoma).

**Table 2:** Main characteristics of the studies included in the meta-analysis.

N	Author	Year	Country	Number of patients	Sensitivity	Specificity	Cutoff point (mAU/mL)	AUROC
1	Young Joon Yoon et al.	2009	Korea	206	0.519	0.97	40	0.777
2	Seung In Seo et al.	2015	Korea	1255	0.739	0.897	40	0.854
3	Xiumei Wang et al.	2017	China	113	0.5221	0.8149	32.09	0.756
4	Shujing Huang et al.	2017	China	163	0.85	0.90	40	0.893
5	Xiao-Qiong Tang et al.	2017	China	366	0.824	0.959	40	0.923
6	Chen et al.	2018	China	459	0.720	0.937	49.5	0.863

**Table 3:** Summary of methodological quality of the included studies on the basis of the review authors' judgments on the items in the QUADAS checklist for each study.

QUADAS	Number					
	1	2	3	4	5	6
Representative patient spectrum?	No	No	No	No	No	No
Selection criteria	Unclear	Yes	Unclear	Unclear	Yes	Yes
Acceptable reference standard?	Yes	Yes	Yes	Yes	Yes	Yes
Acceptable delay between tests?	Yes	Yes	Yes	Yes	Yes	Yes
Partial verification avoided?	Yes	Yes	Yes	Yes	Yes	Yes
Differential verification avoided?	Yes	Yes	Yes	Yes	Yes	Yes
Incorporation avoided?	Yes	Yes	Yes	Yes	Yes	Yes
Index test execution	Yes	Yes	Yes	Yes	Yes	Yes
Reference standard execution	Yes	Yes	Yes	Yes	Yes	Yes
Reference standard results blinded?	Yes	Yes	Yes	Yes	Yes	Yes
Index test results blinded?	Yes	Yes	Yes	Yes	Yes	Yes
Relevant clinical information?	Yes	Yes	Yes	Yes	Yes	Yes
Quality of the studies	A	A	A	A	A	A
